# Milk Yield and Composition of Mixed-Breed Goats on Rangeland during the Dry Season and the Effect on the Growth of Their Progeny

**DOI:** 10.3390/biology10030220

**Published:** 2021-03-13

**Authors:** Manuel J. Flores-Najera, Venancio Cuevas-Reyes, Juan M. Vázquez-García, Sergio Beltrán-López, César A. Meza-Herrera, Miguel Mellado, Luis O. Negrete-Sánchez, Marco A. Rivas-Jacobo, Cesar A. Rosales-Nieto

**Affiliations:** 1 Instituto Nacional de Investigaciones Forestales, Agrícolas y Pecuarias, Campo Experimental La Laguna, Matamoros 27440, Mexico; flores.manuel@inifap.gob.mx; 2 Instituto Nacional de Investigaciones Forestales, Agrícolas y Pecuarias, Campo Experimental Valle de México, Texcoco 56250, Mexico; cuevas.venancio@inifap.gob.mx; 3 Facultad de Agronomía y Veterinaria, Universidad Autónoma de San Luis Potosí, San Luis Potosí 78321, Mexico; manuelvazquez87@yahoo.com (J.M.V.-G.); luis.negrete@uaslp.mx (L.O.N.-S.); marco.rivas@uaslp.mx (M.A.R.-J.); 4 Instituto Nacional de Investigaciones Forestales, Agrícolas y Pecuarias, Campo Experimental San Luis, San Luis Potosí 78431, Mexico; beltran.sergio@inifap.gob.mx; 5 Unidad Regional Universitaria de Zonas Áridas, Universidad Autónoma Chapingo, Bermejillo 35230, Mexico; cmeza2020@hotmail.com; 6 Departamento de Nutrición Animal, Universidad Autónoma Agraria Antonio Narro, Saltillo 25315, Mexico; mmellbosq@yahoo.com; 7 Instituto de Investigación de Zonas Desérticas, Universidad Autónoma de San Luis Potosí, San Luis Potosí 78377, Mexico

**Keywords:** milk composition, milk yield, bodyweight gain, weaning weight

## Abstract

**Simple Summary:**

We tested whether the milk yield capacity and composition of mixed-breed goats on rangeland in northern Mexico during the dry season affects body weight gain and weaning weight of their progeny. Maternal body weight positively influenced milk yield and milk components (*p* < 0.05 to *p* < 0.001). Lactose and solids-non-fat content of milk differed (*p* < 0.05) between goats with different milk yield capacity, whereas milk protein content tended to differ (*p* = 0.08) and milk fat content did not (*p* > 0.05). Milk yield and composition throughout 105-d lactation did not influence body weight gain and weaning weight of the offspring.

**Abstract:**

We tested whether the milk yield capacity of mixed-breed goats on a Chihuahuan desert rangeland in northern Mexico during the dry season affects milk composition, body weight gain, and weaning weight of their progeny. Milk yield and composition, and progeny postnatal growth performance, were recorded weekly. One week after kidding, mixed-breed goats (a mixture of Criollo × dairy breeds; n = 40) were allotted into medium (MP) or low (LP) milk yielding groups (20 goats per group). Mean 105-d total milk yield for MP and LP goats was 45.2 ± 12.5 and 20.7 ± 5.2 L, respectively. Milk lactose (4.3 vs. 4.2%) and solids-non-fat (SNF; 8.2 vs. 8.0) differed (*p* < 0.05) between MP and LP goats; milk protein content tended to differ (*p* = 0.08) between MP and LP goats with no difference for milk fat content (*p* > 0.05). Maternal body weight was positively associated with milk yield, milk lactose, and SNF content (*p* < 0.05 to *p* < 0.001). Goats giving birth to males produce more milk than goats giving birth to females, but milk fat percentage was higher in goats bearing females (*p* < 0.001). Milk yield and composition throughout lactation did not influence body weight gain (47.8 vs. 48.7 g/day for kids from MP and LP goats) and weaning weight (6.7 vs. 6.7 kg from MP and LP goats) of the offspring (*p* > 0.05). Birth weight and weaning weight of the progeny were positively related to maternal body weight (*p* ≤ 0.05). The postnatal growth of the kids was reduced, extending the time to reach market weight. Nevertheless, non-supplemented mixed-breed goats reared on semi-arid rangeland of northern Mexico have the potential for moderate milk production. Therefore, due to the limited nutrients ingested by grazing goats during the dry season, a nutritional supplement is necessary to keep up milk production and adequate growth of kids.

## 1. Introduction

Around the world, goats are distributed across diverse geographical and agro-ecological zones with 95% of them reared in developing countries [1]. Notably, goats are important for milk and meat production and are usually the principal source of income in low-input farming systems [2]. Mexico has more than 8.8 million goats with a predominance of mixed-breed animals, mostly in arid and semiarid ecosystems, that in 2019 contributed to the economy with nearly 40,000 tons of meat (0.7% of the world volume of meat) and 167,000 tons of milk (0.009% of the world volume of milk) [3]. Most of these goats are reared in xerophytic ecosystems, which are characterized by overgrazing, poor quality soils, and low rainfall which makes forage supply highly variable with patterns varying from year to year and with unreliable forage availability [4]. Animals raised under these conditions generally do not receive nutritional supplements because high-quality hay and concentrate feeds are too expensive; therefore, the productivity of the system depends directly on the forage provided by degraded rangelands [5,6].

We have demonstrated that the nutrient content of forages in xerophytic ecosystems in the dry season does not meet the nutritional requirements of gestating and lactating goats [6]. Goats thrive well in xerophytic shrublands because of their particular anatomic characteristics, exceptional browsing ability, and great ability for diet selection [7]. Additionally, goats can include a wide variety of forages rich in secondary compounds in their diet without toxic effects which help them to cope with the nutritional deficiency of the available vegetation [6,8,9]. Thus, goats adapt to terrains with meager forage resources and still are capable of reproducing and producing moderate levels of milk and meat [6,10,11,12,13].

However, limited maternal nutrition during late gestation and early lactation reduced the production and quality of colostrum and milk [14,15], which compromises offspring survival [16]. If offspring survives, postnatal growth is reduced which in turn delays the onset of puberty and decreases reproductive efficiency [17,18,19]. We and others have demonstrated that goats reared on rangelands of northern Mexico selected the same diet irrespective of their milk-yielding potential [6,20]. Yet, milk yield and composition can be manipulated by nutrition and litter size [13,21,22] but a negative relationship between milk quantity and quality exists [23]. Females giving birth to twins produce more milk than females bearing singletons; however, singletons grow faster and are heavier at weaning than twins [22,24]. Nevertheless, production from ruminants in these dryland systems is expected to play an increasingly important role in feeding the world, so innovative and versatile options for livestock production are needed to maximize productivity and improve ecosystem health [25].

Therefore, it was hypothesized that milk composition adds extra value to the growth performance of the progeny under extensive conditions. Thus, milk composition may differ in grazing goats despite their milk-yielding potential. This is important because, in young animals, maternal milk continues to be important and can determine as much as 70% of their growing up to 12 weeks of age [26]. Additionally, a positive relationship between maternal milk production and composition and offspring growth rate exists [27]. Therefore, the objective of the current study was to determine if the milk yield capacity of mixed-breed non-supplemented goats reared in a xerophytic scrub in northern Mexico during the dry season impacts the milk composition, and bodyweight gain and weaning weight of their progeny.

## 2. Materials and Methods

### 2.1. Study Site

The study was conducted on a commercial goat farm on rangeland in northern Mexico (22°39′ N, 101°31′ W, altitude 1850 m). The mean annual temperature is 19 °C (minimal average of 14.1 °C and a maximum average of 21.4 °C) and the average annual precipitation is 334 mm, with 75% occurring from June to October.

### 2.2. Location, and Environmental Conditions

**Figure 1:**
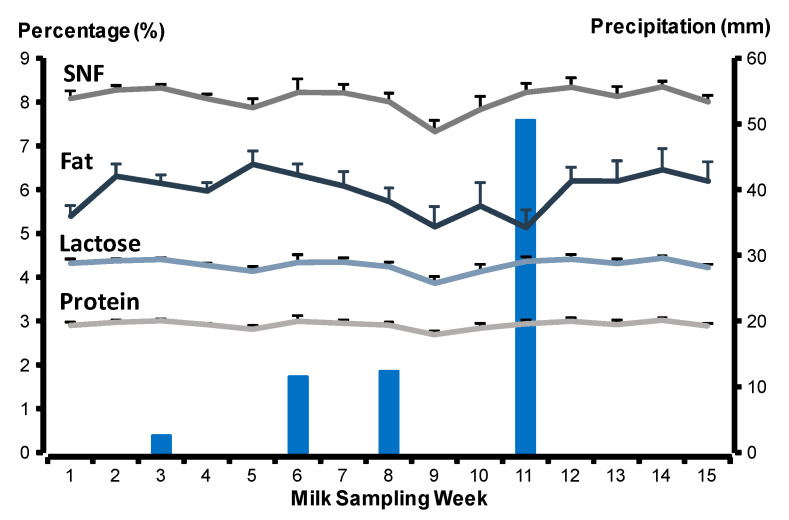
Milk fat, protein, lactose, and solids-non-fat (SNF) from mixed-breed non-supplemented goats reared on a Xerophytic ecosystem in northern Mexico during the dry period (winter–spring). Blue bars represent the precipitation during the study period. The grey dark line represents solids-non-fat content (±SEM), the grey light line represents milk protein content (±SEM), the blue dark line represents milk fat content (±SEM) and the blue light line represents milk lactose content (±SEM).

Environmental data were provided by the Department of Meteorology from the Water National Commission using a meteorology station located 500 m from the place where the goats grassed. Data were recorded daily and include the maximum and minimum environmental temperatures and precipitation. From December to April, the total precipitation was 77 mm (Figure 1). The highest temperature recorded was 33 °C in mid-April (range 20–33 °C) and the lowest temperature was 0 °C in December and January (range 0–11 °C).

### 2.3. Animals, Management, and Response Variables

**Figure 2:**
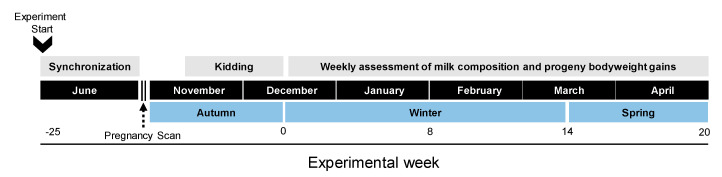
Schematic representation of the main experimental activities performed during the study. Experimental week -25 is when the experiment started and experimental week 20 is when the experiment finished.

To investigate the effect of milk-yielding potential on milk composition and offspring growth, multiparous mixed-breed (Criollo x dairy breeds; mainly French Alpine, Nubian, and Saanen) goats from a commercial herd (n = 150) typical of the extensive farming systems of northern Mexico were used. During the transition into the breeding season (June), ovulation of goats (n = 60) was induced with the insertion of an intravaginal sponge (Chronogest^®^) on day 0, followed by an intramuscular injection of 300 International Units. pregnant mare serum gonadotropin and 50 µg PGF_2_α (Prostaglandin F2α; cloprostenol) on day 9. On day 14, sponges were withdrawn and two days later (day 16), experienced adult bucks were joined to does for 42 days (2 ovarian cycles). Pregnancy and number of fetuses were confirmed by trans-abdominal ultrasonography (Samsung-Medison SA-600; Seoul, Korea; 4 MHz transabdominal convex probe) three times between 45 and 60 days after the onset of joining. Once non-pregnant goats were discarded, 40 goats remained in the study. A week after kidding, goats were allotted into two groups based on their milk production (medium ± SD (772 ± 277 g day^−1^) vs. low ± SD (405 ± 236 g day^−1^)) and were identified accordingly. To set up the groups, milk yield was determined using the oxytocin protocol (more details in Section 2.5, [22,28]). Milk yield gradually increases to reach its peak within 4 to 6 weeks after parturition and accurate forecasting of milk yield can be used in grazing goats within one week after parturition [29]. The mean kidding date (±standard error of the mean (SEM)) was November 30 ± 1.0 d, which corresponds to the end of the rainy season in this area, and the average litter size for medium (MP) and low (LP) milk-yielding groups does was 1.4 ± 0.1. A timeline of activities along the experimental period is shown in Figure 2.

Both groups of goats were permanently maintained together on rangeland from 1000 to 1800 h daily, driven by a herdsman. At night, goats were kept in pens (12 × 10 m) provided with shade and feeders, where they had free access to water and mineral salts containing 17% P, 3% Mg, 5% Ca, and 75% NaCl. We collected data from all the goats until they approached drying (medium ± SD (94 ± 49 g day^−1^) vs. low ± SD (76 ± 59 g day^−1^)). The end of the trial finished with 16 goats from each group. Three goats from MP spontaneously dried off and died and one more spontaneously died. Four goats from LP spontaneously dried off and two of those goats died.

### 2.4. Maternal Body Weight and Offspring Growth

Goat body weight was determined every 15 days, from parturition to week 15 of lactation. Goats were weighed before grazing using a mobile scale with a 200-kg capacity and a precision of 0.05 kg. On the day of kidding, the date, sex, and birth weight of kids were recorded. Offspring body weights were recorded weekly using a mobile scale with a 40-kg weighing capacity and a 0.05-kg readability.

### 2.5. Milk Yield and Composition

One week after parturition from December to April, milk yield and composition were recorded weekly (Figure 2). Goats were milked before grazing on the same day of the week and starting at the same time, using the oxytocin protocol [22,28]. Briefly, goats were separated from their kids the day before milk recording, penned separately, and then hand-milked. To elicit milk let-down, goats received an intramuscular injection of 3 mL of oxytocin (20 IU mg^−1^, PiSA Agropecuaria, Hidalgo; Mexico). After five minutes, goats were milked and the time of the first milking was recorded. Goats were re-milked approximately 3 h later, in the same order as the initial milking, following the same oxytocin protocol. Milk weight collected at the second milking and the time between milkings were recorded to obtain an estimate of milk yield per day. Additionally, in the second milking, a milk sample (10 mL) from each goat was preserved with 0.6 mg mL^−1^ potassium dichromate in a plastic sterile tube and then frozen at −20 °C until milk composition analysis. After the second milking, kids stayed in their corresponding pen and goats went out for grazing. Milk protein, fat, lactose, and solids-non-fat (SNF) were determined using a Milko Tester LTD (MasterEco, Belovo, Bulgaria), after calibration for goat milk according to the manufacturer.

### 2.6. Statistical Analyses

Data were analyzed using the SAS statistical package version 9.3 [30]. Each goat was used as an experimental unit. Maternal live bodyweight change and kid bodyweight gains were fitted in a linear regression model of live weight on time for each individual and the regression coefficient was a measurement of weight change by a unit of time. Bodyweight data were analyzed using mixed linear model procedures and the estimation technique of restricted maximum likelihood (PROC MIXED of SAS). For maternal data, a group of MP or LP goats were considered fixed effects in the model. For offspring data, dam group, birth type, and kid sex were fixed effects in the model. Milk yield, milk components (milk fat, milk protein, milk lactose, solids-non-fat), kid birth weight, bodyweight gain, weaning weight, and maternal weight were included independently as covariates where appropriate.

Milk yield (at 24 h) and milk components (milk fat, milk protein, milk lactose, solids-non-fat) data were analyzed using a mixed linear model (PROC MIXED of SAS). The fixed effect was the dam group according to milk yield level. Environmental variables (precipitation, maximum and minimum environmental temperature), maternal bodyweight, progeny weights (birth weight, lightweight gain, weaning weight), offspring sex, and birth type were included independently as covariates where appropriate. The sampling date was included as a repeated measure. Furthermore, the relationship between two variables (milk yield or milk component (milk fat, milk protein, milk lactose, solids-non-fat) vs. environmental variables or maternal variables or offspring variables) was computed with *LSMeans statement* and the *AT option* (PROC MIXED of SAS), which enables the assignment of arbitrary values to the covariates and determination of if the relationship is positive or negative.

Differences among dates and between maternal groups, litter size, and kids’ sex for bodyweight were analyzed using PROC GLM of SAS. All 2-way interactions among fixed effect and covariates were included in each model and non-significant (*p* > 0.05) interactions were removed from the analysis. For significant differences among treatment means the *LSD option* of SAS was used. Statistical significance was set at *p* < 0.05.

## 3. Results

### 3.1. Maternal Variables

Maternal body weight at the start and end of the experiment did not differ between MP and LP goats (*p* > 0.05; Table 1). Maternal bodyweight changes during the experiment did not differ between groups (*p* = 0.08; Table 1).

### 3.2. Milk Yield

On average, MP goats produced twice as much milk as LP goats (*p* < 0.001; Table 2). Milk yield was influenced by sampling date (*p* < 0.001), milk-yielding potential, and by date × group interaction (*p* < 0.05; Table 2). When groups of goats were combined, maternal body weight was positively associated with milk yield (*p* < 0.01). Milk yield increased 73 g/day as maternal bodyweight increased 5 kg.

Milk yield was positively associated with the sex of offspring (*p* < 0.001; Table 3). Goats rearing male kids produced more milk than goats rearing female kids (*p* < 0.001; Table 3). Birth weight influenced positively milk yield (*p* < 0.001; Table 3) but not daily bodyweight gain or weaning weight (*p* > 0.05; Table 3). Environmental variables (minimal temperature, maximum temperature, and precipitation) did not affect milk yield (*p* > 0.05; Table 3).

### 3.3. Milk Composition

*Milk fat content.* Milk fat content did not differ between MP and LP goats (*p* > 0.05; Table 2) but differed among sampling dates (*p* < 0.05; Figure 1). Minimal temperature influenced positively (*p* < 0.05), maximum temperature influenced negatively (*p* < 0.05) and precipitation had no effect on milk fat content (*p* > 0.05). Milk fat content differed between goats suckling male or female kids (*p* < 0.001); dams bearing females produced 0.9 percentage points more milk fat than dams bearing males. Milk fat content was positively related to the daily weight gain of offspring (*p* < 0.001); milk fat content increased by 0.37% as bodyweight gain increased by 20 g. Milk fat content was not influenced by birth weight or weaning weight of kids (*p* > 0.05). Maternal body weight was negatively associated with milk fat content (*p* < 0.001; Table 3).

*Milk protein content.* Milk protein content tended to differ between milk-yielding groups (*p* = 0.06; Table 2). Milk protein content remained steady across the experimental period and did not differ among sampling dates (*p* > 0.05; Figure 1). Milk protein content was not influenced by precipitation, minimal and maximum temperature, offspring variables (sex, birth weight, bodyweight gains, and weaning weight), and maternal body weight (*p* > 0.05; Table 3).

*Milk lactose content.* Milk lactose content remained stable across the experimental period; it differed between MP and LP goats (*p* < 0.05; Table 2), but not with sampling dates (*p* > 0.05; Figure 1). Milk lactose content was positively influenced by maternal body weight (*p* < 0.001); milk lactose content increased by 0.23% as maternal body weight increased 10 kg. Milk lactose content was not influenced by precipitation, minimal and maximum temperature, sex, birth weight, bodyweight gains, and weaning weight of the kids (*p* > 0.05; Table 3).

*Solids-non-fat (SNF).* SNF content remained steady across the experimental period and differed between MP and LP goats (*p* < 0.05; Table 2) and did not differ among sampling dates (*p* = 0.08; Figure 1). SNF content was positively related to maternal body weight (*p* < 0.05); SNF content increased 0.36% as maternal body weight increased 10 kg. SNF content was not influenced by precipitation, minimal and maximum temperature, sex, birth weight, bodyweight gains, and weaning weight of kids (*p* > 0.05; Table 3).

### 3.4. Birth Weight and Growth Performance of Kids

Kid birth weight averaged 2.7 ± 0.1 kg, with no difference between MP and LP goats (*p* > 0.05; Table 4). Birth weight was similar between single-born or twin-born kids (*p* > 0.05; Table 4). Birth weight differed between kid’s sex, with males being heavier than females at birth (*p* < 0.05; Table 4).

The average daily bodyweight gain was 48.3 ± 2.7 g day^−1^ with no difference for kids whose dams had medium or low milk yield or sex of kids (*p* > 0.05; Table 4). On average, kids born as singletons grew faster than those born as twins (*p* < 0.01). Kid weaning weight averaged 6.7 ± 0.2 kg. Progeny weaning weight did not differ between MP and LP goats or sex of kids (*p* > 0.05; Table 4). On average, kids born as singletons were heavier at weaning than those born as twins (*p* < 0.05).

Bodyweight gains and weaning weight of the kids were not influenced by milk yield or milk composition of their dams across the experimental period (*p* > 0.05). Birth weight and weaning weight were positively related to maternal body weight (*p* ≤ 0.05), whereas progeny daily bodyweight gain was not related to maternal body weight (*p* = 0.08). Birth weight increased 0.1 kg as maternal bodyweight increased 5 kg, whereas weaning weight increased 0.2 kg as maternal bodyweight increased 5 kg.

## 4. Discussion

We previously demonstrated that goats with different milk yield potential selected the same diet in xerophytic ecosystems [6], but whether milk yield production of mixed-breed goats on semi-arid rangeland affects milk composition remains inconclusive. Despite the dry conditions prevailing during the study, a marked difference in milk yield between groups of goats persisted throughout lactations. This indicates that the herd had a highly heterogeneous genetic structure in terms of the milk yield capacity of goats. Furthermore, milk lactose, protein, and SNF were higher in MP than in LP goats, whereas milk fat content did not differ between MP and LP goats. Simos et al. [31] reported that milk fat, protein, and SNF were negatively correlated with increased milk yield in goats, and Mmbengwa et al. [32] found a negative correlation between SNF and milk yield. This is contrary to our finding where medium milk yield did not depress milk components in mixed-breed goats. Our results, however, are in line with the results of Idamokoro et al. [33] who found no correlation between milk yield and milk compositions for non-descript grazing goats.

Throughout the experiment, the percentage of most milk components remained steady. Daily weight gain of kids was positively influenced by milk fat content but not by the rest of the milk components, whereas weaning weight was not influenced by any milk component tested. We further observed that milk composition was influenced by maternal body weight, except milk protein content. Nevertheless, it seems that milk composition is affected by milk yield level despite consumption of the same diet on rangeland.

Our first hypothesis was that milk composition differs in goats with different milk yields. Differences in milk yield were consistent throughout lactation; yet, the percentage of milk lactose and protein were higher in MP goats. Different authors indicated that milk protein and lactose content can be manipulated by modifying the protein source in the diet but not the milk yield [34,35,36], whereas milk fat content is negatively related to milk yield [13,37]. The milk composition reported in the present experiment is within the range reported previously in grazing goats [13,38]. Extending these observations, goats bearing male kids produced more milk than goats bearing female kids but the percentage of milk fat content was higher in those goats giving birth to female kids. The negative relationship between milk yield and milk fat content can be explained simply by the negative genetic correlation between milk yield and milk constituents [39] or the dilution effect and a decrease in fat mobilization [37]. Nevertheless, these observations collectively indicate that nutrition and milk-yielding potential can modify the milk composition in mixed-breed goats on rangeland.

Furthermore, we observed that maternal bodyweight and offspring birth weight were positively associated with milk yield, whereas maternal bodyweight was negatively associated with milk fat content. Previously it was reported that heavier goats at mating or gestation produced heavier progeny at birth [22,40,41], and larger fetuses produced more placental lactogen leading to a positive relationship between birth weight and milk yield [22,42]. Additionally, heavy females produce more milk than do their lighter counterparts [22], which could be because heavier kids at birth consume more milk, thus stimulating milk production and promoting mammary gland development [43,44]. This explains the differences in growth between singletons and twins and between males and females [22,45]. Indeed, milk yield is positively correlated to litter size, and goats suckling twins produce more milk than those suckling singletons [22] yet we were not able to confirm these observations. Failing to meet the nutritional requirements for lactation reduced the maternal lactational capacity [14,15,46]; therefore, the conditions under which the experiment was conducted, and the reduced available vegetation may have substantially contributed to the similar milk yield observed between goats suckling twins or singletons [6]. Nevertheless, these observations warrant more research under extensive conditions because bigger and heavier animals are not always the most efficient [47].

Moreover, we observed that milk fat content was influenced by environmental factors but not milk yield or milk content of protein, lactose, and SNF. We observed that milk fat content increased as the minimum temperature increased, whereas milk fat content decreased as the maximum temperature increased. Extending our results, goats kidding in winter increased their milk yield and milk fat and protein [48]. Conversely, hot environments and high environmental temperatures resulted in increased rectal temperature and reduced milk fat content [49,50].

Our second hypothesis was that milk composition influences the growth performance of kids. We observed that the daily bodyweight gains and weaning weight were not influenced by milk composition; therefore, we reject our second hypothesis. Similarly, Rosales-Nieto et al. [22] indicated that milk composition is not a limiting factor for offspring growth, whereas dam milk yield and genetic background are important for offspring growth; yet, in the current experiment, milk yield of the dam was not related to progeny growth. Furthermore, daily bodyweight gains and weaning weight did not differ between MP or LP goats. Moreover, birth weight differed between male and female kids; however, growth performance was similar between them, indicating a possible catch-up growth in female kids [51]. In the current experiment, singletons grew faster than twins, confirming previous observations [22,24,45].

Notably, the daily bodyweight gains of kids averaged 48 g and the weaning weight 6.7 kg. Similar to our results, Gaytan et al. [52] reported low daily weight gain in goat kids reared under extensive conditions (67 g), indicating that goat kids are forced to ingest native forage at an early age. In extensive systems in northern Mexico, the main objective of goat farmers is to obtain milk for 4 to 6 months, while the second objective is to rear goat kids for meat. Furthermore, small ruminants represent the principal economic output, contributing a large share of the farmer’s income. Indeed, weight and size at birth are determined not only by their genetic potential but also by maternal environment and non-genetic factors [22,45,53]. Yet, low birth weight reduced growth performance, thus resulting in low meat potential (yield and quality) and low market-selling weight [54,55,56]. Nevertheless, goats on rangeland during the dry season should receive a nutritional supplement to ensure sufficient milk yield for their kids and to reach an adequate market weight of kids [57].

## 5. Conclusions

We concluded that moderate milk yield is possible with mixed-breed goats in winter on rangeland without feed supplementation. Genotypes of goats with medium milk yield potential presented higher milk lactose, protein, and SNF, whereas milk fat content was similar between MP and LP goats. Milk yield increased as maternal body weight and birth weight increased; yet, milk yield and composition throughout lactation did not influence bodyweight gain and weaning weight of kids. These results reaffirm that mixed-breed goats grazing on xerophytic shrubland of northern Mexico can thrive in extremely harsh environments, and are capable of producing moderate milk production even in winter, with scarce forage and low nutrient availability.

## Figures and Tables

**Table 1:** Maternal body weight at the beginning and end of the experimental period and bodyweight change during lactation from goats at medium and low milk yielding potential.

Variable	Medium Milk Yield	Low Milk Yield	SEM	*p*-Value
BW beginning lactation (kg)	35.56	35.06	2.80	0.78
BW end lactation (kg)	33.14	34.16	3.80	0.71
Bodyweight change (g/day)	−37.8	−23.8	11	0.08

SEM = Standard error of the mean; BW = bodyweight.

**Table 2:** Milk yield and composition (fat, protein, lactose, solids-non-fat) from non-supplemented mixed-breed goats reared on a Xerophytic ecosystem in northern Mexico during the dry season (winter–spring) with different milk yielding potentials (medium vs. low).

Variable	Medium Milk Yield	Low Milk Yield	SEM	*p*-Value
Milk yield (g day^−1^)	423	203	56	0.001
Milk fat (%)	5.8	6.0	0.25	0.15
Milk protein (%)	3.0	2.9	0.05	0.06
Milk lactose (%)	4.3	4.2	0.08	0.02
Solids-non-fat (%)	8.2	8.0	0.15	0.02

SEM = Standard error of the mean.

**Table 3:** The relationship among milk composition (fat, protein lactose, and solids-non-fat) and environmental conditions, progeny growth variables, and maternal body weight from non-supplemented goats reared on a Xerophytic ecosystem in northern Mexico during winter–spring with different milk yielding potentials (medium vs. low). The data from goats with different milk yielding potentials are combined.

Variable	Prec	Tmax	Tmin	SexOff	BirthW	BWgain	WeanW	MoBW
Milk yield (g day^−1^)	NS	NS	NS	***	***	NS	NS	***
Fat (%)	0.07	*	*	***	NS	***	NS	**
Protein (%)	NS	NS	NS	NS	NS	NS	NS	NS
Lactose (%)	NS	NS	NS	NS	NS	NS	NS	***
Solids-non-fat (%)	NS	NS	NS	NS	NS	NS	NS	*

Abbreviations: Prec = precipitation; Tmax = maximum temperature; Tmin = minimal temperature; SexOff = sex of the offspring; BT = birth type (singleton or wtin); BirthW = birth weight; BWgain = bodyweight gains; WeanW = weaning weight; MoBW = maternal body weight. *p* value: * *p* < 0.05; ** *p* < 0.01; *** *p* < 0.001; NS: not significant. Data are presented across the milk-yielding potential of dams.

**Table 4:** Birth weight (BWT), average daily bodyweight gain (BWG), and weaning weight (WWT) of kids from mixed-breed non-supplemented goats reared on a Xerophytic ecosystem in northern Mexico during the dry season (winter–spring) with different milk yield potentials (medium vs. low).

		BWT (kg)	BWG (g day^−1^)	WWT (kg)
Milk yield	Medium	2.9	47.8	6.7
	Low	2.6	48.7	6.7
SEM		0.33	7.7	0.7
Sex	Female	2.5 ^a^	44.6	6.4
	Male	2.9 ^b^	52	7.0
SEM		0.27	7.4	0.7
Birth type	Singleton	2.7	57.7 ^a^	7.5 ^a^
	Twin	2.7	43.6 ^b^	6.3 ^b^
SEM		0.32	7.1	0.7

Data of milk-yielding potential of goats across sex and birth type of kids. Data of sex of the kids are presented across the milk-yielding potential of dams and birth type. Data of birth type are presented across the milk-yield potential of dams and sex of kids. SEM = Standard error of the mean. Within columns and variables, means with different superscripts differ (*p* < 0.05).

## Data Availability

Not applicable.

## Software and Data Repository Resource

The data presented in the manuscript are not deposited in an official repository.
